# Supplementary material for: HMGB1 Promotes In Vitro and In Vivo Skeletal Muscle Atrophy through an IL-18-Dependent Mechanism
Source: Cells. 2022 Dec 6;11(23):3936. doi: 10.3390/cells11233936 (PMC9740799; doi:10.3390/cells11233936)
Supplement: Supplementary file 1 [file cells-11-03936-s001.zip › cells-2038245-supplementary.pdf]

Supplementary Tables

Table S1. List of primers used in qRT-PCR

**Gene**	**Sequences (5'-3')**
Atrogin-1	Forward: GAGTGGCATCGCCCAAAAGA<br>Reverse: TCTGGAGAAGTTCCCGTATAAGT
MuRF-2	Forward: GTCCTGGTGACACAGATTGGAT<br>Reverse: TGCTGCCTATGTGCTTCTCA
Myostatin	Forward: CTGGTCCTGGGAAGGTTACA<br>Reverse: ACGCTACCACGGAAACAATC
IGF-1	Forward: GGACCGAGGGGCTTTTACTT<br>Reverse: GTGGGGCACAGTACATCTCC
MyoD	Forward: GAGGATCCGATGGAGCTTCTATCG<br>Reverse: CGGATCCTCTCAAAGCACCTGATA
Pax-7	Forward: GGTCCCCAGGATGATGAGA<br>Reverse: TTGATGAAGACCCCACCAAG
MyoG	Forward: GACCTGATGGAGCTGTATGAG<br>Reverse: CTGAAGGTGGACAGGAAGG
IL-18	Forward: GACCTGGAATCAGACAACTTTGG<br>Reverse: GCCTCGGGTATTCTGTTATGGA
IL-6	Forward: GATGGTCTTGGTCCTTAGCC<br>Reverse: GGGAAATCGTGGAAATGAGA
IL-1β	Forward: GCAACTGTTCCTGAACTCAACT

	Reverse: ATCTTTTGGGGTCCGTCAACT
TNF-α	Forward: CAAAGGGAGAGTGGTCAGGT<br>Reverse: GGCAACAAGGTAGAGAGGC
HMGB1	Forward: GCA TCC TGG CTT ATC CAT TG<br>Reverse: TGC TCT TTT CAG CCT TGA CC
GAPDH	Forward: ACCACAGTCCATGCCATCAC<br>Reverse: TCCACCACCCTGTTGCTGTA

Table S2. List of reagents, resources, and antibodies.

**ANTIBODIES / REAGENTS / RESOURCES**			
**ANTOBODIES**	**HOST/ISOTYPE**	**SOURCE**	**IDENTIFIER**
GDF-11/GDF-8 polyclonal antibody	Goat / IgG	R&D system	Cat#MAB788-SP
MURF2 polyclonal antibody	Goat / IgG	Thermo Fisher Scientific	Cat#PA5-18142
IGF-1 polyclonal antibody	Rabbit / IgG	Thermo Fisher Scientific	Cat#PA5-27207
Neutralizing RAGE polyclonal antibody	Rabbit / IgG	Thermo Fisher Scientific	Cat#PA1-075
Myosin skeletal muscle polyclonal antibody	Rabbit / IgG	Thermo Fisher Scientific	Cat#PA5-31466
MAFbx/Atrogin-1 (F-9) monoclonal antibody	Mouse / IgG	Santa Cruz Biotech	Cat#sc-166806
MyoD (G-1) monoclonal antibody	Mouse / IgG	Santa Cruz Biotech	Cat#sc-377460
PI3-kinase p85α (B-9) monoclonal antibody	Mouse / IgG	Santa Cruz Biotech	Cat#sc-1637
Akt1 (B-1) monoclonal antibody	Mouse / IgG	Santa Cruz Biotech	Cat#sc-5298

p-Akt1/2/3 (Thr308) monoclonal antibody	Rabbit / IgG	Santa Cruz Biotech	Cat#sc-16646-R
c-Jun (G-4) monoclonal antibody	Mouse / IgG	Santa Cruz Biotech	Cat#sc-74543
p-c-Jun (KM-1) monoclonal antibody	Mouse / IgG	Santa Cruz Biotech	Cat#sc-822
HMG-1/HMGB1 (J2E1) monoclonal antibody	Mouse / IgG	Santa Cruz Biotech	Cat#sc-135809
Neutralizing IL-18 monoclonal antibody	Rat / IgG	MBL	Cat#D048-3
IL-18 polyclonal antibody	Rabbit / IgG	Abcam	Cat#ab71495
Pax-7 polyclonal antibody	Rabbit / IgG	Abcam	Cat#ab187339
Dystrophin polyclonal antibody	Rabbit / IgG	Abcam	Cat#ab15277
Desmin polyclonal antibody	Rabbit / IgG	Abcam	Cat#ab216616
α-Tubulin monoclonal antibody	Mouse / IgG	Abcam	Cat#ab7291
Phospho-PI3 kinase p85(Tyr458)/p55(Tyr199) polyclonal antibody	Rabbit / IgG	Cell Signaling Tech	Cat#4228S
mTOR (7C10) monoclonal antibody	Rabbit / IgG	Cell Signaling Tech	Cat#2983S
Phospho-mTOR (Ser2448) (D9C2) monoclonal antibody	Rabbit / IgG	Cell Signaling Tech	Cat#5536S
β-Actin monoclonal antibody	Mouse / IgG	Sigma-Aldrich	Cat#A5441
Rabbit anti-goat IgG-HRP conjugated secondary antibody	Rabbit /IgG	Sigma-Aldrich	Cat#A8919
Goat anti-rabbit IgG-HRP conjugated secondary antibody	Goat /IgG	Santa Cruz Biotech	Cat#sc-2004

Mouse anti-rabbit IgG-HRP conjugated secondary antibody	Mouse / IgG	Santa Cruz Biotech	Cat#sc-2357
Goat anti-rabbit IgG (H+L), Alexa Fluor 488 conjugated secondary antibody	Goat / IgG	Thermo Fisher Scientific	Cat#A32731
Goat anti-rabbit IgG (H+L), Alexa Flour 594 conjugated secondary antibody	Goat / IgG	Thermo Fisher Scientific	Cat#A32740
**CHEMICALS, siRNA, PLASMIDS, AND RECOMBINANT PROTEIN**		**SOURCE**	**IDENTIFIER**
Recombinant mouse HMGB1protein		Biorbyt	Cat#orb57027
LY294002 (PI3 kinase inhibitor)		Enzo Life Sciences	Cat#ALX-270-038
Akt inhibitor		Sigma-Aldrich	Cat#A6730
Rapamycin (mTOR inhibitor)		Sigma-Aldrich	Cat#R0395
Tanshinone IIA		Santa Cruz Biotech	Cat#sc-200932
Control siRNA		Dharmacon Research	Cat#D-001810-10-05
P85 siRNA		Dharmacon Research	Cat#L-041079-00-0005
Akt siRNA		Dharmacon Research	Cat#L-040709-00-0005
mTOR siRNA		Dharmacon Research	Cat#L00300800
RAGE siRNA		Santa Cruz Biotech	Cat#sc-36374
c-Jun siRNA		Santa Cruz Biotech	Cat#sc-29223
Mouse shRNA HMGB1 plasmid		National RNAi Core Facility (Sinica, Taiwan)	Cat#TRCN0000365913
CMV promoter plasmid		Addgene	Cat#169739
MD plasmid		Addgene	Cat#20864
DAPI		Sigma-Aldrich	Cat#D9564
Glycerol solution		Sigma-Aldrich	Cat#56-81-5

**CRITICAL COMMERCIACL ASSAY**	**SOURCE**	**IDENTIFIER**
Mouse IL-18 ELISA Kit	R&D systems	Cat#DY7625-05
IHC Kit	Sigma-Aldrich	Cat#00-4955-58
**CELLS LINE**	**SOURCE**	**IDENTIFIER**
C2C12 mouse myoblast	ATCC	Cat#CRL-1772
G7 mouse myoblast	ATCC	Cat#CRL-1447
293 T	Thermo Fisher Scientific	Cat#R700007
**CULTURE MEDIUM**	**SOURCE**	**IDENTIFIER**
Dulbecco's Modified Eagle's Medium (DMEM)	Gibco	Cat#41966-029
Fetal bovine serum (FBS)	Gibco	Cat#21875-034
Horse serum (HS)	Gibco	Cat#16050-122
Trypsin	Gibco	Cat#15090-046
**MOUSE MODEL**	**SOURCE**	**IDENTIFIER**
C57BL/6J mice	The Jackson Laboratory	Cat#000664
**SOFTWARE**	**SOURCE**	**IDENTIFIER**
ImageJ software	Wayne Rasband (NIH)	https://imagej.nih.gov/ij/
GraphPad Prism version 8.2.1	GraphPad	https://www.graphpad.com/
BioRender software	BioRender	https://biorender.com/
**OTHER**		
Immobilon Western Chemiluminescent HRP substrate	Merck millipore	Cat#WBKLS0500

Supplementary Figures

Supplementary Figure S1

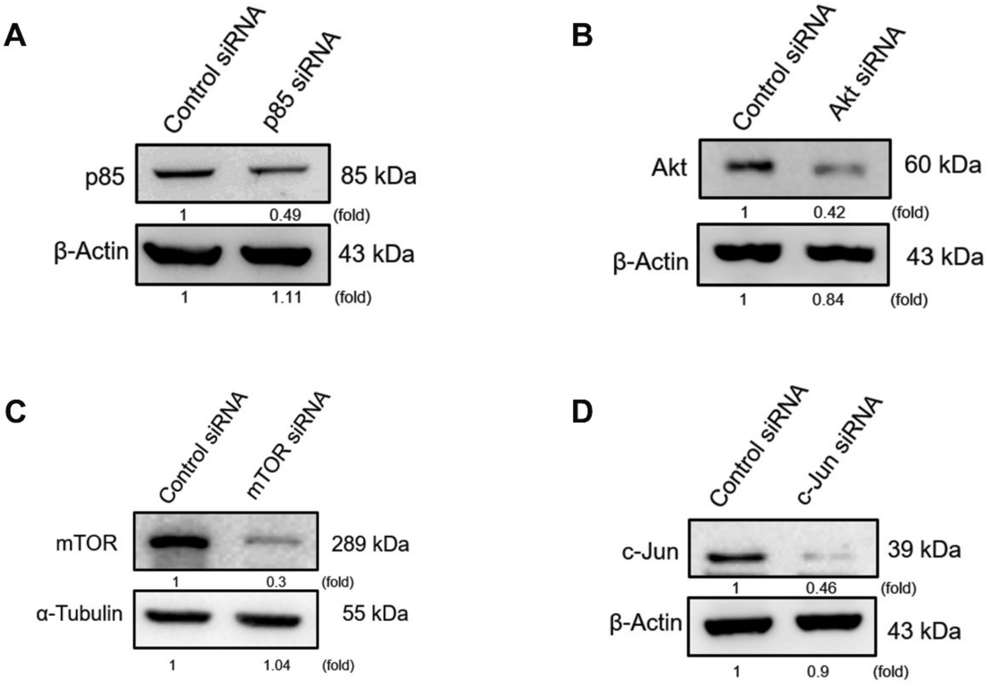

Supplementary Figure S1. Western blot detects the knockdown efficacy of various siRNAs against p85, Akt, mTOR, and c-Jun molecules. C2C12 cells were transfected with control siRNA or (**A**) p85 siRNA, (**B**) Akt siRNA, (**C**) mTOR siRNA, and (**D**) c-Jun siRNA for 24 h. Total proteins were then detected by immunoblotting. β-Actin was used as a loading control. ImageJ software was used to quantify the immunoblotting results and normalized with β-Actin.

Supplementary Figure S2

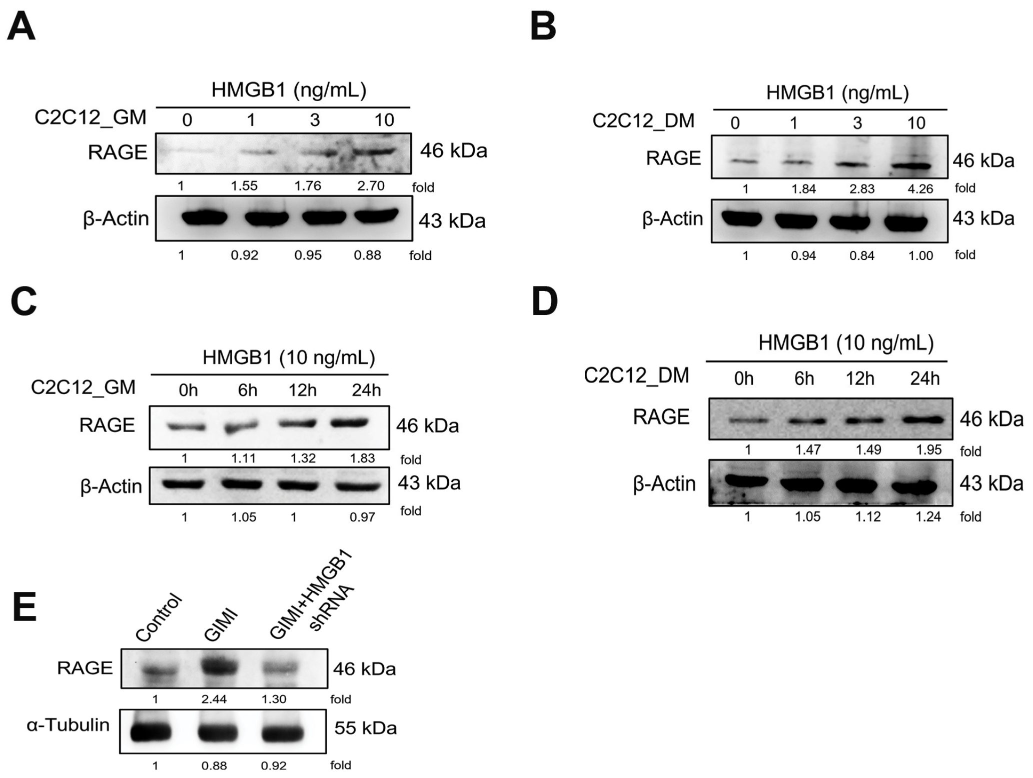

Supplementary Figure S2. Western blot detects the expression of RAGE protein expression following HMGB1 treated with dose-dependent manner into C2C12 myoblast cells in growth medium (**A**) or differentiation medium (**B**). Western blot detects the expression of RAGE following HMGB1 (10 ng/mL) treated with time-dependent manner into C2C12 myoblast cells in the growth medium (**C**) and differentiation medium (**D**). β-Actin was used as a loading control. The RAGE expression in glycerol-induced muscle injury (GIMI) mouse model (**E**). α-Tubulin was used as a loading control. ImageJ software was used to quantify the immunoblotting results and normalized with the loading control.

Supplementary Figure S3

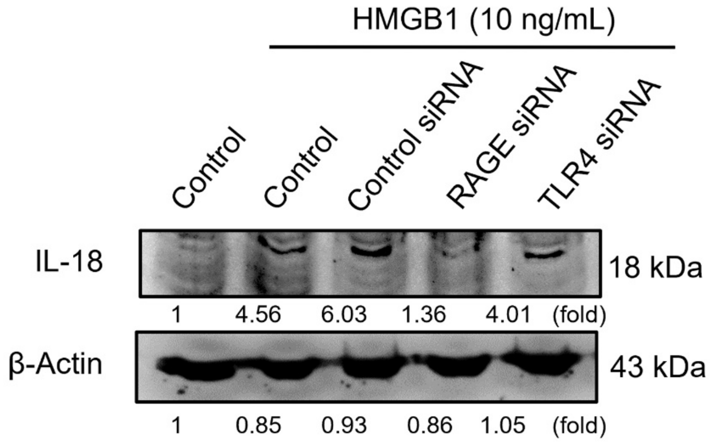

Supplementary Figure S3. Western blot detects the expression of IL-18 for siRNAs against RAGE or TLR4 following HMGB1 treatment in C2C12 cells. β-Actin was used as a loading control. ImageJ software was used to quantify the immunoblotting results and normalized with β-Actin.

Supplementary Figure S4

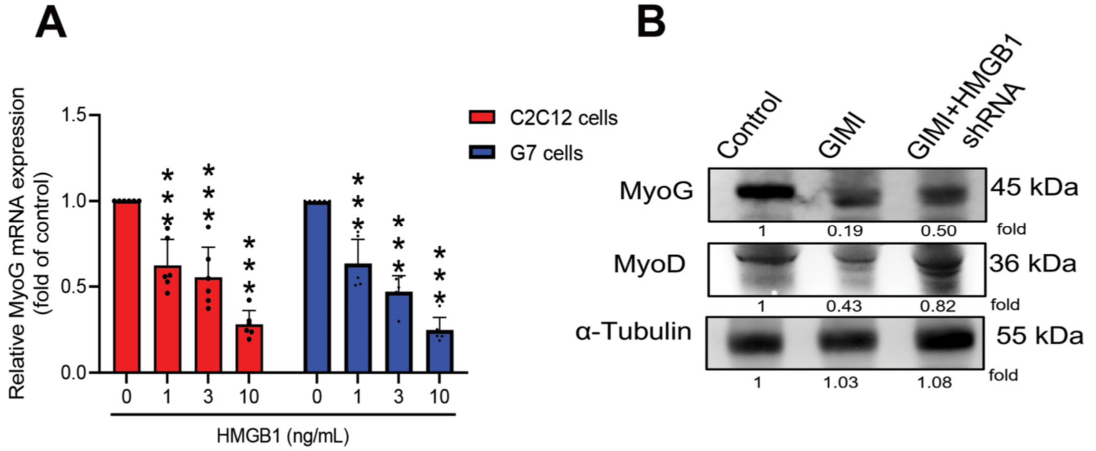

Supplementary Figure S4. The expression of myogenic markers *in vitro* and *in vivo*. (**A**) HMGB1 induced myogenin (MyoG) expression with dose-dependent manner in murine C2C12 and G7 cells lines (n = 6). (**B**) The expression of MyoG and MyoD was detected by Western blot in a glycerol-induced muscle injury (GIMI) mouse model (n = 3). α-Tubulin was used as a loading control. ImageJ software was used to quantify the immunoblotting results and normalized with the loading control. Results are expressed as means ± SD. Statistical analysis was done by using one-way ANOVA and represented as *p* value. $^{*}p < 0.05$ compared with the control group.
